# A new species of *Uralaphorura* Martynova, 1978 from northeast China, with a key to world species of the genus (Collembola, Onychiuridae)

**DOI:** 10.3897/zookeys.662.12071

**Published:** 2017-03-21

**Authors:** Zhijing Xie, Xin Sun, Wanda Maria Weiner

**Affiliations:** 1 Key laboratory of Wetland Ecology and Environment, Northeast Institute of Geography and Agroecology, Chinese Academy of Sciences, Changchun 130102, China; 2 University of Chinese Academy of Sciences, Beijing 100049, China; 3 J.F. Blumenbach Institute of Zoology and Anthropology, University of Göttingen, 37073 Göttingen, Germany; 4 Institute of Systematics and Evolution of Animals, Polish Academy of Sciences, Sławkowska 17, 31-016 Kraków, Poland; 5 Xin Sun

**Keywords:** Chaetotaxy, taxonomy

## Abstract

A new species, *Uralaphorura liangshuiensis* **sp. n.**, has been reported from Xiao Hinggan Mountains (northeast China). The new species is unique in having the pso formulae as 32/133/33332 dorsally and 0/000/0001(0) ventrally, and the ventral psx formula as 3/000/221000. A key to all known species of the genus is given.

## Introduction

Martynova (1976) established *Uralia* as a subgenus of *Onychiurus* Gervais, 1841 for *O. schilovi* Martynova, 1976. In 1978, she proposed a new name, *Uralaphorura*, instead of *Uralia*, as the former name has been used for a genus of birds. Later, Weiner (1996) raised the subgenus to generic level. The genus is characterized by having simple or slightly bilobed vesicles in PAO, chaeta d0 present on the head, eleven chaetae in the distal whorl of tibiotarsi, and a triangular arrangement of the anterior cephalic pseudocelli (Babenko 2009). To date, there are four known species belonging to the genus *Uralaphorura*: *U. schilovi* (Martynova, 1976) from northern Europe: *U. varicellata* Babenko, 2009, *U. yanensis* Babenko, and *U. tunguzica* Babenko, 2007 from Siberia. The latter was recently also recorded from China (Sun and Wu 2013).

Based on a study of specimens collected from Xiao Hinggan Mountains (northeast China), a new species is reported and a key to all known worldwide species is given.

## Materials and methods

Specimens were collected by hand using a brush, cleared in lactic acid and then mounted in Marc André II solution. They were studied with a Nikon Eclipse 80i microscope.

Labial type is named after Fjellberg (1999). Labium areas and chaetal nomenclature follow Massoud (1967) in D’Haese’s (2003) modification. Chaetae on anal valves are named following Yoshii (1996). Chaetae on the furcal area are classified in accordance with Weiner (1996). The formulae of pseudocelli, parapseudocelli and pseudopores are their number on half-tergum (dorsally) or half-sternum (ventrally). The description of tibiotarsal chaetotaxy is based on Deharveng’s (1983) scheme and is expressed as a total number of chaetae and also number of chaetae in whorls C, B, and A+T, for example: 22 (3, 8, 11).

### Abbreviations used in the description

**Abd.** abdominal segments

**Ant.** antennal segments

**AIIIO** ensory organ of Ant. III

**d0** unpaired axial chaeta on area frontalis on head

**ms** s-microchaaeta (microsensillum)

**PAO** postantennal organ

**p-chaeta** chaeta of row p

**pso** pseudocellus

**psp** pseudopore

**psx** parapseudocellus

**Th.** thoracic segments

**1^m^** unpaired pseudopore.

## Taxonomy

### Uralaphorura liangshuiensis sp. n.

Taxon classificationAnimaliaCollembolaOnychiuridae

http://zoobank.org/79E65A21-008F-49E2-8770-D13529F63FC6

Figs 1 , 2

#### Material Examined.

Holotype female, China, Heilongjiang Province, Yichun City, Liangshui National Park (47°11'04"N, 128°53'00"E, alt. 347m), 18 June 2016, under moss on the dead-and-down wood, Xin Sun, Zhijing Xie, Wanda M. Weiner and Grzegorz Paśnik leg. Paratypes, 17 females and 9 males, same data as holotype.

The holotype and 23 paratypes (15 females and 8 males) are deposited in Northeast Institute of Geography and Agroecology, Chinese Academy of Sciences; 3 paratypes (2 females and 1 male) in Institute of Systematics and Evolution of Animals, Polish Academy of Sciences.

**Figure 1.**
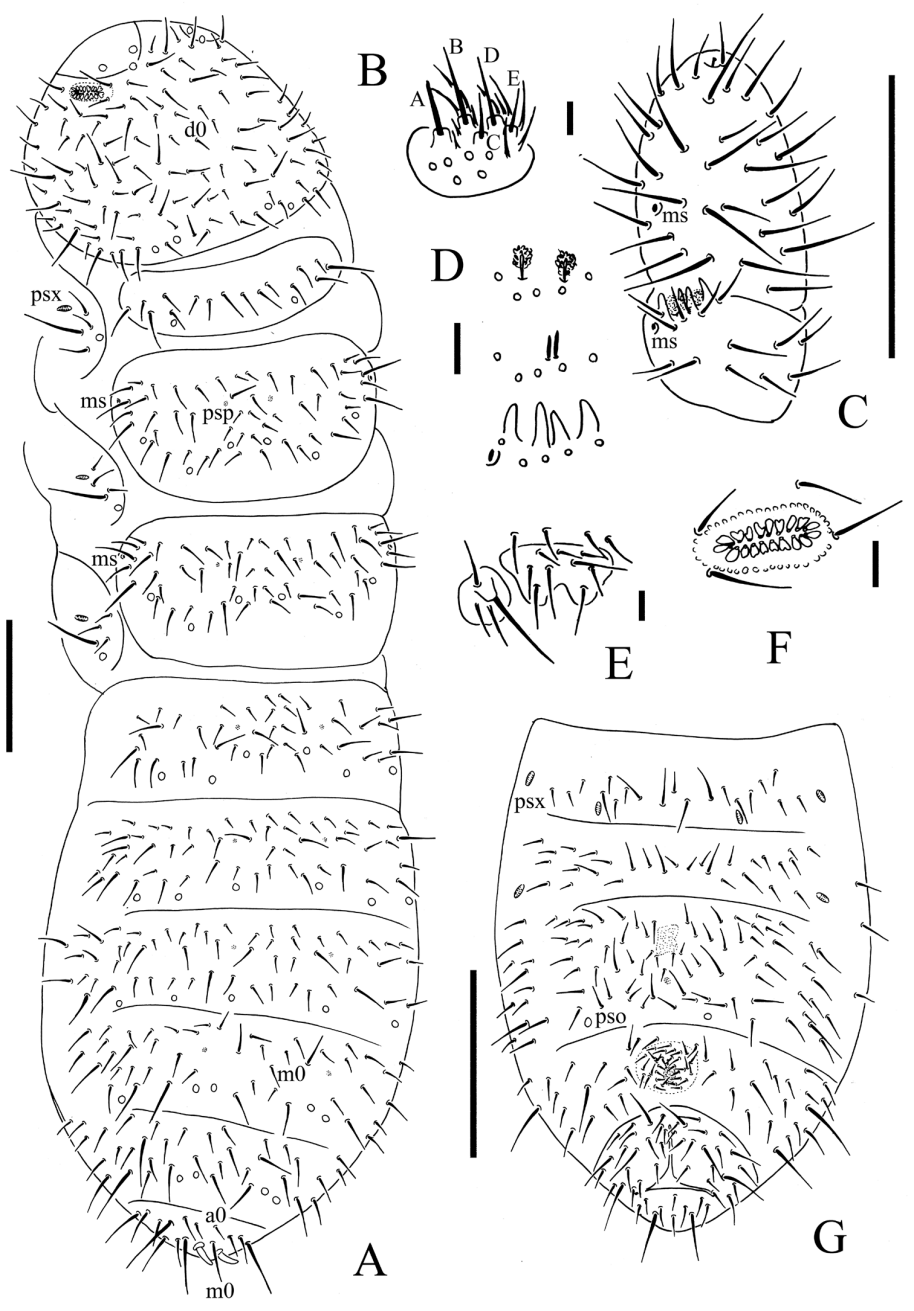
*Uralaphorura liangshuiensis* sp. n. **A** dorsal side of body **B** labium **C** Ant. III–IV **D** clubs, rods and papillae of AIIIO **E** labrum and maxillary palp **F** PAO **G** ventral side of Abd. II–VI. Scale bars: 0.1 mm **A, C, G**; 0.01 mm **B, D–F**.

**Diagnosis.** Pso formulae 32/133/33332 dorsally and 0/000/0001(0) ventrally; psx only present ventrally, formula as 3/000/2210(1)00; subcoxae 1 of legs I–III with 1 pso and 1 psx each; AIIIO consists of four papillae, five guard chaetae, two small rods, and two granulated sensory clubs; 3+3 p-chaetae present between two medial pso posterior part of head; Abd.IV tergum with axial chaeta m0, Abd.V tergum without unpaired axial chaeta, Abd.VI tergum with axial chaetae a0 and m0; anal spines 0.7 times as long as inner edge of hind unguis.

**Description.** *Body* white in alcohol. Length of body 1.1–1.7 mm in females, 0.9–1.1 mm in males; holotype 1.6 mm. Shape of body cylindrical with anal spines on papillae. Anal spines 0.7 times as long as inner edge of hind unguis.

*Pso formulae* 32/133/33332 dorsally and 0/000/0001(0) ventrally (Figs 1A, G). Psx only present ventrally, formula as 3/000/2210(1)00 (Figs 1A, G). Two of all available specimens have 1+1 psx on ventral Abd. IV instead of 1+1 pso. Subcoxae 1 of legs I–III with 1 pso and 1 psx each. Psp formula 00/011/111100 dorsally and 00/111/0001^m^0 ventrally (Fig. 1A, G).

**Figure 2.**
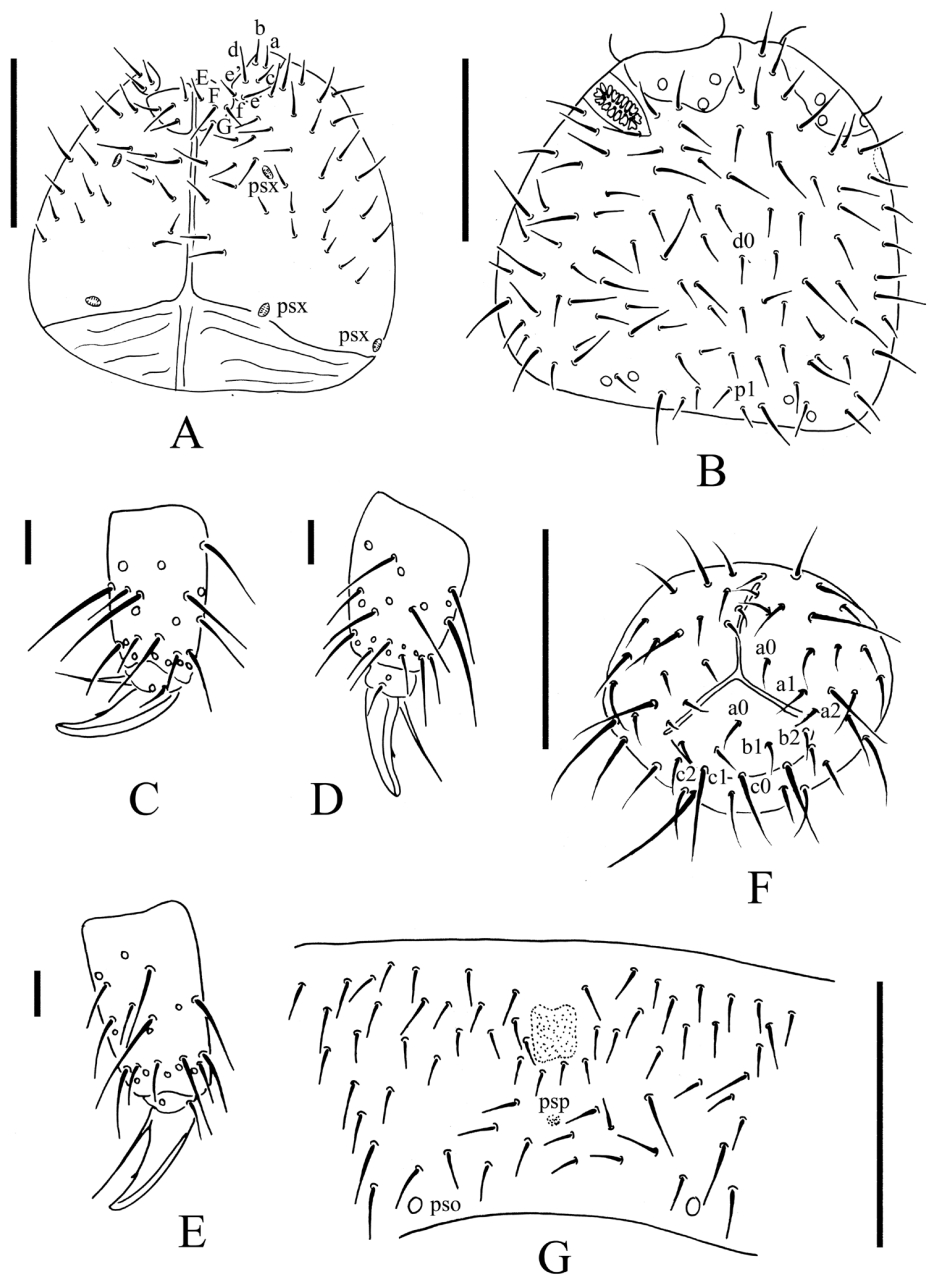
*Uralaphorura liangshuiensis* sp. n. **A** ventral side of head **B** dorsal side of head; **C**, distal part of leg I **D** distal part of leg II **E**, distal part of leg III; F, anal valves **G** Abd. sternum IV with furcal remnant. Scale bars: 0.1 mm **A–B, F–G**; 0.01 mm **C–E**.

*Head*. Antennae as long as head. Length ratio of Ant. I: II: III: IV as approximately 1: 1.5: 1.5: 2.5. Subapical organite on Ant. IV with globular apex; basolateral ms above the second proximal row of chaetae (Fig. 1C).AIIIO consists of four papillae, five guard chaetae, two small rods, and two granulated sensory clubs (Fig. 1C); lateral ms just behind sensory organ. Ant. II with 17–18 chaetae. Ant. I with nine chaetae. Antennal base well marked. PAO with 16–20 simple or slightly bilobed vesicles arranged in two rows along axis of organ (Fig. 1F). Dorsal cephalic chaeta d0 present (Fig. 2B). On head 3+3 p-chaetae present between two medial pso posterior part of head, p1 anterior to others (Fig. 2B). Mandible with strong molar plate and four apical teeth. Maxilla bearing three teeth and six lamellae. Maxillary palp simple with one basal chaeta and two sublobal hairs (Fig. 1E). Labral chaetae 4/342 (Fig. 1E). Labium with six proximal, four basomedian (E, F, G, and f) and six basolateral (a, b, c, d, e, e’) chaetae (Figs 1B, 2A); labial type A, papillae A–E with 1, 4, 0, 3 and 3 guard chaetae respectively (Fig. 1B). 4+4 postlabial chaetae present along ventral groove (Fig. 2A).

*Body chaetotaxy*. S-chaetae not distinguishable from ordinary chaetae. Tiny and blunt ms present on both Th. II and III (Fig. 1A). Th. I tergum with 8–10+8–10 chaetae. Th. II–III terga with 3–4+3–4 chaetae along axial line respectively (Fig. 1A). Abd. I–III terga with 4+4 chaetae along axial line respectively (Fig. 1A). Abd. IV tergum with axial chaeta m0, Abd.V tergum without unpaired axial chaeta, Abd. VI tergum with axial chaetae a0 and m0 (Fig. 1A). Th. I–III sterna without chaetae.

*Appendages*. Subcoxae 1 of legs I–III with 4, 4 and 4 chaetae. Tibiotarsi of legs I, II and III with 22 (3, 8, 11), 23 (4, 8, 11) and 22 (4, 7, 11) chaetae (Fig. 2C, D, E).Unguis with teeth on inner edge. Unguiculus as long as inner edge of unguis, without inner basal lamella (Fig. 2C, D, E). Ventral tube with 7–8+7–8 distal chaetae and 1–2+1–2 basal chaetae, without anterior chaetae. Furca reduced to finely granulated area, with 4 small dental chaetae arranged in one row posteriorly; one manubrial row of chaetae present (Fig. 2G).

*Female genital plate* with 17–21 chaetae, males with 30–42 chaetae. Male ventral organ absent. Anal valves with numerous acuminate chaetae; each lateral valve with chaetae a0, 2a1, 2a2; upper valve with chaetae a0, 2b1, 2b2, c0, 2c1 and 2c2 (Fig. 2F).

#### Ecology.

Under moss on dead-and-down wood in the Korean pine forest.

#### *Derivatio nominis*.

Named for the national park in which the species was found.

#### Discussion.

The new species can easily be distinguished from all known congeners due to the unique number of dorsal and ventral pso. Thus, it shares the presence of ventral pso only with *U. varicellata*, but clearly differs from the latter in their number (0/000/0001(0) in the new species vs 2/000/0112 in *U. varicellata*). Dorsal pso formula of the new species is most similar although not identical to that of *U. tunguzica* (32/133/33333 vs 32/133/33332 in *U. liangshuiensis* sp. n.). Existed differences in ventral psx formulae (3/000/2210(1)00 in the new species, 2/000/221200 in *U. schilovi*, 2/000/221100 in *U. tunguzica*, 2/000/210000 in *U. varicellata*, and 2/000/221200 in *U. yanensis*) can also be used in separation of the known species (see also key below).

### Key to the known species of the genus *Uralaphorura*

**Table:** 

1	Pseudocelli on Th. I absent	**2**
–	Pseudocelli on Th. I present	**3**
2	Unpaired axial chaeta on Abd. VI tergum present, tibiotarsi with clavate chaetae	***U. schilovi* (Martynova, 1976)**
–	Unpaired axial chaeta on Abd. VI tergum absent, tibiotarsi without clavate chaetae	***U. yanensis* Babenko, 2009**
3	Number of pseudocelli on Abd. V tergum as 2, number of parapseudocelli on ventral head as 3	***U. liangshuiensis* sp. n.**
–	Number of pseudocelli on Abd. V tergum as 3, number of parapseudocelli on ventral head as 2	**4**
4	Ventral pseudocelli absent, subcoxae with one pso and one psx	***U. tunguzica* Babenko, 2007**
–	Ventral pseudocelli formula as 1/000/0112, subcoxae with 2–3 pso	***U. varicellata* Babenko, 2009**

## Supplementary Material

XML Treatment for Uralaphorura liangshuiensis
